# Supplementary material for: The effect of intrapartum prolonged oxygen exposure on fetal metabolic status: secondary analysis from a randomized controlled trial
Source: Front Endocrinol (Lausanne). 2023 Jun 27;14:1204956. doi: 10.3389/fendo.2023.1204956 (PMC10335765; doi:10.3389/fendo.2023.1204956)
Supplement: Supplementary file 3 [file DataSheet_3.zip › Result-X101SC21092977-Z01-J001-B1-42/2.MetAnnotation/Lipidmaps/meta_pos.Lipidmaps.Anno.pdf]

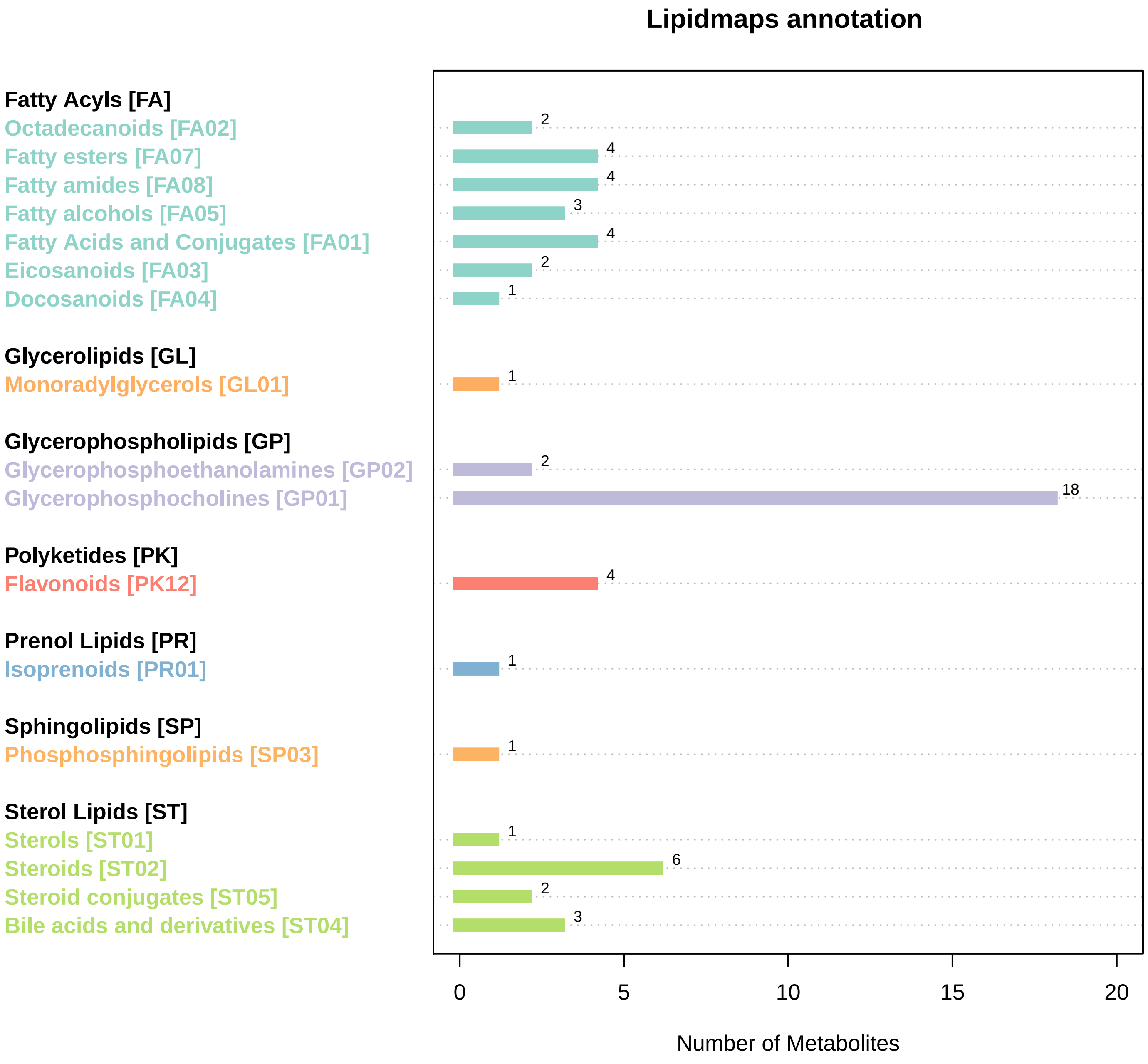

Lipidmaps annotation
Fatty Acyls [FA]
Octadecanoids [FA02]
Fatty esters [FA07]
Fatty amides [FA08]
Fatty alcohols [FA05]
Fatty Acids and Conjugates [FA01]
Eicosanoids [FA03]
Docosanoids [FA04]
Glycerolipids [GL]
Monoradylglycerols [GL01]
Glycerophospholipids [GP]
Glycerophosphoethanolamines [GP02]
Glycerophosphocholines [GP01]
Polyketides [PK]
Flavonoids [PK12]
Prenol Lipids [PR]
Isoprenoids [PR01]
Sphingolipids [SP]
Phosphosphingolipids [SP03]
Sterol Lipids [ST]
Sterols [ST01]
Steroids [ST02]
Steroid conjugates [ST05]
Bile acids and derivatives [ST04]
2
4
4
3
4
2
1
1
2
18
4
1
1
1
6
2
3
0
5
10
15
20
Number of Metabolites
